# Obstructive sleep apnea in adults with Down syndrome: body composition, metabolic profile and cognitive status

**DOI:** 10.1016/j.clinsp.2026.100867

**Published:** 2026-02-13

**Authors:** Lígia Fidelis Ivanovic, Cristiana Castanho de Almeida Rocca, Rosa Hasan, Pâmella Pollyanna Braga Carra Costela, Geraldo Lorenzi-Filho, Orestes Vicente Forlenza, Milton A. Martins, Patricia Zen Tempski

**Affiliations:** a Hospital das Clínicas da Faculdade de Medicina da Universidade de São Paulo, São Paulo, SP, Brazil; b Instituto de Psiquiatria do Hospital das Clínicas da Universidade de São Paulo, São Paulo, SP, Brazil; c Centro de Desenvolvimento de Educação Médica (CEDEM) da Faculdade de Medicina da Universidade de São Paulo, São Paulo, SP, Brazil; d Instituto do Coração do Hospital das Clínicas da Faculdade de Medicina da Universidade de São Paulo, São Paulo, SP, Brazil

**Keywords:** Down syndrome, Sleep apnea, Obstructive, Body composition, Cognition, Memory

## Abstract

**Context:**

Down Syndrome (DS) is the main genetic cause of Intellectual Disability (ID) and is characterized by the presence of several medical comorbidities. Among these, Obstructive Sleep Apnea (OSA) stands out, with a prevalence much higher than that of the general population. The clinical impact of OSA in subjects without DS described in the literature includes increased cardiovascular, metabolic and neurocognitive risk. However, among the adult population with DS, these associations are poorly described.

**Objectives:**

To evaluate the presence and characterize OSA in adults with DS and its association with body composition, metabolic profile, functional and cognitive performance.

**Methods:**

This cross-sectional study included 34 persons with DS aged 18 years or over. The measurements were: anthropometry, complete Polysomnography in the Sleep laboratory (PSG), neuropsychological assessment using the Abbreviated Wechsler Intelligence Scale and NEUPSILIN Brief Assessment Instrument, biochemical assessments, and functionality scales (Barthel and Lawton). There were 30 representative PSG. Subjects with and without OSA were compared regarding clinical, anthropometric, functional (*n* = 30), and cognitive (*n* = 20) variables. Sleep changes were correlated with the variables under study.

**Results:**

The frequency of OSA (apnea-hypopnea index ≥ 5 events/h) was 63 %. As compared to no OSA, OSA was associated with male sex (*p* = 0.001), greater cervical circumference (34 vs. 40 cm, *p* < 0.001), greater deposition of abdominal fat (82 vs. 92 cm, *p* = 0.049), higher average levels systolic blood pressure (*p* = 0.03), higher frequency of altered fasting blood glucose or glycated hemoglobin equal to or greater than 5.7 % (*p* = 0.03) and low HDL cholesterol (*p* = 0.004). OSA was associated with worse performance in episodic and semantic verbal memory (*p* = 0.02).

**Conclusion:**

Adults with DS have a high frequency of OSA, which may be related to metabolic profile and worse memory performance.

## Highlights

• Sleep apnea in Down syndrome may relate to obesity and body fat distribution.
• Abdominal fat and neck size are linked to sleep apnea in Down syndrome.
• Sleep apnea may be related to metabolic changes in adults with Down syndrome.
• Obstructive sleep apnea may impair memory and cognition in Down syndrome.
• Obstructive sleep apnea in Down syndrome is linked to poor memory performance.

## Introduction

Down Syndrome (DS) is the main genetic cause of Intellectual Disability (ID).^1^ The prevalence of DS among live births varies between studies from different countries, with rates estimated at 4:10.000 in Southeastern Brazil.^2^ The life expectancy of patients with DS increased in recent decades, reaching an average of 60-years, due to technical progress in medical interventions and their early implementation, notably heart surgery, in addition to better living conditions and advances in healthcare.^3^ Adults with DS have an increased incidence of many medical comorbidities higher than that of the general population, including obesity, visual and auditory deficits, epilepsy, hypothyroidism and celiac disease.^4^, ^5^, ^6^, ^7^ With their increased life expectancy, it is becoming clear that the early onset of dementia is a major challenge in this population.

Obstructive Sleep Apnea (OSA) is the most common sleep-disordered breathing and is characterized by the collapse of the Upper Airways (UA), generating episodes of reduction or cessation of airflow, corresponding to hypopneas or apneas, respectively. The repetitive respiratory events cause sleep fragmentation and intermittent hypoxia. Studies on the prevalence of OSA in adults with DS vary widely from as low as 9.56 % to 100 % due to methodological variations, including definition of respiratory events and use of simplified methods for OSA diagnosis.^5^^,^^8^, ^9^, ^10^

Obesity, metabolic syndrome, male gender, hypothyroidism, acromegaly, increased volume of tonsillar and adenoid tissues, and craniofacial abnormalities such as retrognathia and maxillary hypoplasia are risk factors for OSA.^11^ Obesity is a major cause of narrowing of the UA.^12^ Obesity is associated with the deposition of adipose tissue in the neck structures with a consequent increase in cervical circumference, which in turn increases the risk of OSA.^13^ People with DS are at increased risk of having OSA. In addition to the higher prevalence of hypothyroidism and obesity, they have characteristic anatomical changes that result in a reduction in the size of the airway at rest and greater airway collapsibility, including maxillary hypoplasia, relative macroglossia and generalized hypotonia.^14^ OSA is associated with metabolic effects that include increased insulin resistance and the risk of developing diabetes mellitus; cardiovascular diseases, including increased activity of the sympathetic autonomic system, endothelial dysfunction with consequent systemic arterial hypertension and increased risk of cardiovascular events (arrhythmias, stroke, coronary artery disease). OSA is also associated with neurological impairment, worsening of memory consolidation; psychological disorders with a greater propensity to changes in mood, concentration and fatigue.^15^^,^^16^

The clinical impact of OSA in subjects without DS described in the literature includes increased cardiovascular, metabolic, and neurocognitive risk.^11^^,^^17^ However, among the adult population with DS, these associations are poorly described,^18^ as is their relationship with functional performance, which is crucial for independent and autonomous participation in community life. Therefore, the aim of the present pilot, exploratory study was evaluate the presence and characterize Obstructive Sleep Apnea in adults with DS Down syndrome and its possible associations with body composition, metabolic profile and cognitive performance.

## Methods

### Participants and study design

This is a pilot, exploratory, cross-sectional study that followed the criteria of the Strengthening the Reporting of Observational studies in Epidemiology (STROBE). Participants came from the outpatient clinic for comprehensive care for persons with Down syndrome of the Discipline of General Practice and Propaedeutic of the Clinical Hospital of the Faculty of Medicine of the University of São Paulo (HCFMUSP), which began in February 2018, based on referrals from Lapa rehabilitation institute, specialty clinics at HCFMUSP and basic health units.

The inclusion criteria were: being 18-years or older, not having any decompensated comorbidities, including epilepsy, heart, thyroid, psychiatric diseases, suspected visual and/or auditory deficit without investigation and treatment, and also, being able to walk without an aid device. Subjects with hypothyroidism, visual or hearing alterations must be clinically compensated and using prosthetics (when indicated) to participate in all stages of the study.

All participants and their legal guardians received information regarding the study protocol and signed an informed consent form before participation. The Research Ethics Committee of the Clinical Hospital of the Faculty of Medicine of the University of São Paulo approved the study (CAAE: 23,969,319.8.0000.0068 number 3.686.083).

### Measurements

#### Body composition

Weight was measured using a previously calibrated digital scale (Filizola® ‒ capacity up to 200 kg), in an orthostatic position, wearing light clothes. Height was measured with a wall-mounted stadiometer, Sanny® (range of 80 cm to 220 cm), in an upright position, barefoot, with arms extended along the body and head. The Body Mass Index (BMI) was calculated by dividing weight in kilograms by height in squared meters.

Abdominal Circumference (AC) was measured at the midpoint between the last rib and the iliac crest, with an inelastic tape (cm), with the patient in an upright position, at the end of expiration. The AC/height was calculated by dividing the AC by height in centimeters.

Cervical Circumference (CC) was determined with an inelastic tape (cm) positioned above the cricoid cartilage and perpendicular to the long axis of the neck, measured in a sitting position.

#### Clinical diagnosis for metabolic conditions

Participants with altered values in the serum biochemical analysis, in two or more measurements, separated in time, during the study follow-up, were considered to have:

• Glycemic change if fasting blood glucose ≥ 100 mg/dL and/or HbA1c ≥ 5.7 %.
• Low HDL if ≤ 40 mg/dL.
• Isolated hypercholesterolemia if LDL ≥ 160 mg/dL.
• Hypertriglyceridemia if triglycerides ≥ 150 mg/dL.
• Hyperuricemia if uric acid ≥ 7 mg/dL.
• Overweight if Body Mass Index (BMI) 25 to 29.99 kg/m^2^ and obesity if BMI ≥ 30 kg/m^2^.

Subjects with clinical or subclinical hypothyroidism, diagnosed previously or during the study period, were classified as hypothyroid, using the reference from the laboratory of the central institute of HCFMUSP (free T4 0.7‒1.48 ng/dL; TSH 0.35–4.94 µIU/mL).

#### Daily functioning

Instrumental activities of daily living were assessed using the Lawton scale, adapted version recommended by the Brazilian Ministry of Health in 2007. The scale assesses the skills of: using the telephone, going to distant places without special planning, shopping, preparing meals, tidying up the house, doing small home repairs, washing and ironing one's clothes, taking medication at the correct dose and time, and taking care of finances. Basic activities of daily living were assessed using the Barthel scale. The Barthel index consists of ten items: bowel control, bladder control, grooming, toilet use, feeding, transfer, walking, dressing, stair climbing, and bathing.^19^ All questionnaires applied were answered with the help of their main and responsible caregiver at the time of the assessment.

#### Cognitive assessments

Neuropsychologists performed the cognitive assessments. The abbreviated Wechsler Intelligence Scale (WASI) was applied, consisting of four subtests: vocabulary (four items in the form of a figure and 38-items represented by words), cubes (use of colored cubes to produce two-color figures), similarities (identifying similar figures or explain how two objects or concepts are similar) and matrix reasoning (where a part of a picture is missing and the examinee must say which part is missing) in order to determine the total IQ, verbal IQ (vocabulary and similarity subtests) and IQ execution (subtests of cubes and matrix reasoning).^20^ The Neupsilin Brief Assessment Instrument, developed in Brazil in 2008 by Fonseca, Salles and Parente, was also applied, which assesses attention skills, temporo-spatial orientation, visual perception, memory, arithmetic skills, language, praxis and executive functions. For statistical analysis, the NEUPSILIN subtests most affected in OSA were selected, using the *Z*-score (individual standardized).^21^

#### Polysomnography

The participants performed a polysomnogram with the Biologic or Brain Wave III digital polygraph in the Clinical Neurophysiology bed of the Institute of Psychiatry at HCFMUSP, with the simultaneous and continuous performance of the following parameters: electroencephalogram, electro-oculogram, submental and anterior tibial electromyogram, snoring sensor, nasal and oral airflow, nasal pressure transducer, thoracic and abdominal respiratory effort, peripheral oxygen saturation, body position and one-lead electrocardiogram. The polysomnograms were evaluated according to the criteria established by the American Academy of Sleep Medicine. Obstructive apneas were defined as a reduction of 90 % or more in airflow despite ventilatory effort, lasting at least 10 s, during sleep. Hypopnea was defined as a reduction of 30 % or more in the flow, for at least ten seconds, associated with a desaturation of 3 % or more in peripheral oximetry, or arousal due to ventilatory effort detected by thoracoabdominal movement.

Results were expressed as: Total Sleep Time (TST), sleep efficiency; Apnea and Hypopnea Index/hour of sleep (AHI); sleep stages (Rapid Eye Movement ‒ REM and non-REM), Apnea and Hypopnea Index/hour of sleep in REM and non-REM stages, AHI in the supine position.

### Data collection period and assessment schedule

For the analysis of the prevalence of comorbidities, diagnoses made between February 2018 and February 2023 were considered.

Anthropometric data were evaluated in 2 moments: on the same day as the application of the socio-demographic and functionality questionnaires, with this part of the protocol always being carried out on the same week as the cognitive assessment (*n* = 24) and at the nearest appointment (before or after) polysomnography (*n* = 34).

For the assessment of the presence and characterization of OSA, all participants with polysomnography performed during the study period or before it were included in the analysis, regardless of the time it was performed, provided that the patient had completed at least 3 h of sleep. In cases where >1 polysomnography was performed, the most recent exam was selected.

For the comparison analyzes between subject with and without OSA and for the correlation analyzes of OSA with body composition, participants who underwent polysomnography within the maximum interval of 15-months (before or after the physical and cognitive protocol), ensuring the absence of any relevant clinical, functional or anthropometric change, in addition to 2 subjects with polysomnography performed 30- and 44-months before who did not present significant clinical and anthropometric change at follow-up, were included.

### Statistical analysis

The data were analyzed using the R program, version 4.3.1. The variables were analyzed for normal distribution using the Shapiro-Wilk Test. Quantitative variables were expressed as mean ± standard deviation or median (interquartile range 25 %‒75 %) according to their parametric distribution or not, respectively. Qualitative variables were expressed in percentages.

Variables with a parametric distribution were tested with the t-test to compare means after analysis of variance with the *F* test. Non-parametric variables were compared with the Wilcoxon test. For correlation analyses, Pearson's tests were used when parametric and Spearman's test when non-parametric. The assessment of the association between qualitative variables was performed using the Chi-Square or Fisher test, when appropriate. All tests applied were two-tailed, and the significance level adopted was 5 % and a confidence interval of 95 %.

## Results

**Fig. 1:**
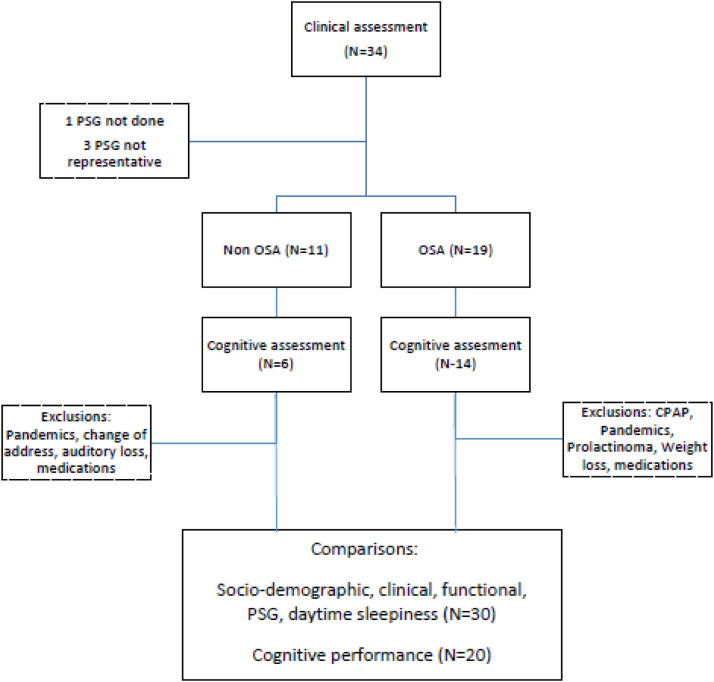
Study flowchart.

Of the total test group included (*n* = 34), 24 subjects underwent cognitive assessment during the study period. Of these 24, four of them were excluded from the comparative analysis between OSA and cognitive performance, namely: one who did not undergo polysomnography for operational reasons, two who had non-representative polysomnography, and one patient who underwent polysomnography three years after the cognitive assessment and there was a significant clinical change in the time interval that elapsed. The other 10 subjects did not undergo the cognitive assessment due to issues related to treatment, clinical complications, change of address, and the COVID-19 pandemic (Fig. 1).

For the clinical description, 34 subjects were included, with a median age of 24 years (IQR 25‒75: 22‒28), 53 % male, 50 % of these were literate and had a median of 9-years of education (IQR 25‒75: 9‒12). The average body mass index was 28.58 kg/m^2^ ± 5.2, with an average abdominal circumference/height ratio of 0.60 and normal blood pressure levels (Systolic blood pressure 108±10 mmHg; Diastolic blood pressure 61±6 mmHg). Their mean abdominal circumference was 90±12.76 cm, and the cervical circumference mean was 38±3.59 cm.

Despite being young adults, a high percentage of clinical comorbidities was found (Table 1). Specific ophthalmological and otorhinolaryngological pathologies, such as keratoconus and hearing loss, found with high frequency, highlight the importance of periodic evaluation with these specialties. Other clinical conditions in the various organs and systems also showed high prevalence, with emphasis on lipid changes, overweight, obesity, hypothyroidism, celiac disease, gastroesophageal reflux disease, renal lithiasis, cervical instability, and obstructive sleep apnea.

**Table 1:** Clinical assessment.

**Ophthalmological (*****n*****= 34)**	**% (n)**
Refraction error	91.18 (31)
Cataract	8.82 (3)
Keratoconus	11.76 (4)
lacrimal tract disease	5.88 (2)
**Otorhinolaryngological (*n* = 34)**	**% (n)**
Hearing loss	32.35 (11)
Rhinitis	29.41 (10)
Adenoidectomy	14.7 (5)
Tonsillectomy	26.47 (9)
Palatoplasty	2.94 (1)
Ventilation tube	11.76 (4)
Obstructive sleep apnea	63.33 (19/30)
**Endocrinometabolic (*n* = 34)**	**% (n)**
Altered fasting blood glucose or glycated hemoglobin ≥ 5.7 %	23.53 (8)
Low HDL	41.18 (14)
Isolated hypercholesterolemia	14.71 (5)
hypertriglyceridemia	32.35 (11)
Mixed hypercholesterolemia	8.82 (3)
Hyperuricemia	41.18 (14)
Hipothyroidism	73.53 (25)
Overweight	44.12 % (15)
Obesity	32.36 % (11)
**Cardiologic (*n* = 34)**	**% (n)**
Arrhythmia	2.94 (1)
Interatrial septal defect (ASD)	32.35 (11)
Ventricular septal defect (VSD)	20.59 (7)
Tetralogy of Fallot	2.94 (1)
Patent ductus arteriosus	2.94 (1)
Valvular heart disease	8.82 (3)
Cardiac surgery	26.47 (9)
**Gastroenterological (*n* = 34)**	**% (n)**
Celiac disease	11.76 (4)
Chronic calculous cholecystopathy	17.65 (6)
Constipation	8.82 (3)
Gastroesophageal Reflux Disease	26.47 (9)
Liver disease	8.82 (3)
Steatosis	43.75 (7/16)
**Nefrourological (*n* = 34)**	**% (n)**
Renal lithiasis	23.53 (8)
**Neuropsychiatric (*n* = 34)**	**% (n)**
Anxiety	17.65 (6)
Depression	5.88 (2)
Epilepsy	2.94 (1)
Schizophrenia	5.88 (2)
Obsessive compulsive symptoms	2.94 (1)
Autism spectrum disorder	5.88 (2)
Nocturnal agitation	8.82 (3)
Movement disorder	2.94 (1)
**Orthopedic (*n* = 34)**	**% (n)**
Scoliosis	14.71 (5)
Cervical instability	19.35 (6/31)
**Medication (*n* = 34)**	**% (n)**
Anticonvulsant	5.88 (2)
Antidepressant	29.41 (10)
Statin	8.82 (3)
Proton pump inhibitor	20.59 (7)
Levothyroxine	70.59 (24)
Metformin	2.94 (1)
Neuroleptic	20.59 (7)

As regards the sleep study, 34 polysomnograms were performed during the study period, which were well tolerated by the majority of participants. Of these, three were not included in this analysis because they did not represent a usual night's sleep, with 38-, 83- and 129-minutes of total sleep time, respectively, and one patient who was unable to perform due to the pandemic, totaling 30 polysomnographies considered representative. Considering these, the prevalence of OSA was 63.3 %. The study participants had reduced sleep efficiency, with an average of 72.94 % and a significant reduction in the percentage of REM sleep (average 10.23 %), with values greater than or equal to 85 % being expected for efficiency and between 20 % and 25 % for REM sleep. The apnea-hypopnea index had a median of 8.85 events per hour, with higher values during REM sleep, reaching a median of 30.2 events per hour of sleep. For most of the time, subjects slept in the supine position (median 79 % vs. 21 % non-supine time), and their AHI in the supine position was higher with a median of 12.5 events/hour (Table 2).

**Table 2:** Polysomnography (*n* = 30).

Variable	
TST (min)	330.64 ± 70.44
Eficiency ( %) (normal ≥ 85 %)	72.94 ± 14.64
TTS REM sleep ( %) (20 %‒25 %)	10.23 ± 6.46
AHI (event/hour)	8.85 (3.5; 26.8)
AHI in REM sleep (event/hour)	30.2 (2.87; 54.9)
AHI in non-REM sleep (event/hour)	7.4 (1.45; 26.07)
AHI in supine (DD) (event/hour)	12.5 (3.82; 31.67)
AHI non supine	2.4 (0.9; 6.35)
Time in supine position ( %)	79 (49.2; 100)
Time in non-supine position ( %)	21 (0; 50.75)

Parametric variables expressed as means ± standard deviation. Non-parameric variables expressed as medians and interquartile range (25‒75).

TST, Total Sleep Time; AHI, Apnea Hipopnea Index; REM, Rapid Eye Moviment.

Participants with AHI ≥5, regardless of the presence of daytime symptoms, were compared with participants without OSA, with respect to sociodemographic, anthropometric, clinical, functional (Table 3) and polysomnographic variables (Table 4). In this analysis, 30 subjects who had undergone polysomnography were considered representative and included. There was no statistically significant difference regarding age, education in years, or literacy. There was a statistically significant association between the presence of apnea and the male sex. The subject diagnosed with OSA had greater cervical circumference (*p* < 0.001) and greater abdominal fat deposition (*p* = 0.049). The effect size of the difference between groups for cervical circumference was large (g Hedge = 2.211) as well as for abdominal circumference (g Hedge = 0.78). In this sample, subjects with male sex had lower BMI as they were taller (male BMI 27.4 kg/h^2^ vs. female 29.1; male height 1.57 m vs. female 1.45; *p* < 0.001), which may explain the lack of association, in this study, between BMI and AHI.

**Table 3:** OSA × no-OSA: socio-demographic, clinical, functional and daytime sleepiness.

Variable	AHI < 5 (*n* = 11)	AHI ≥ 5 (*n* = 19)	p
Age (years)	26.72 ± 7.12	26.36 ± 6.73	0.89
Male sex	9.1 % (1)	73.68 % (14)	**0.002**
Literacy ( %)	63.6	47.4	0.47
BMI (kg/m^2^)	26.69 ± 3.74	29.11 ± 6.11	0.24
AC (cm)	82.54 ± 9.35	92.61 ± 14.45	**0.049**
AC/height	0.56 ± 0.05	0.60 ± 0.1	0.21
CC (cm)	34 (33. 35)	40 (39.41)	**<0.001**
Glycemic change % (n)	0 (0)	31.57 (6)	**0.036**
Hypertriglyceridemia % (n)	18.18 (2)	31.57 (6)	0.672
Low HDL ( %)	0 (0)	52.63 (10)	**0.004**
Hypercholesterolemia % (n)	0 (0)	21.05 (4)	0.268
Hyperuricemia % (n)	18.18 (2)	47.36 (9)	0.14
Hypothyroidism % (n)	72.72 (8)	68.42 (13)	>0.99
SBP (mmHg)	102 ± 10	110 ± 9	**0.03**
DBP (mmHg)	61 ± 6	61 ± 7	0.80
Epworth	4.9 ± 5.46	7.55 ± 5.38	0.21
Nap % (n)	11.1 (1)	44.4 (8)	0.193
Barthel index (points)	100 (97.5; 100)	95 (90; 100)	0.18
Lawton (points)	15.18 ± 3.7	15 ± 4.17	0.90

Parametric variables expressed as means ± standard deviation. Non-parameric variables expressed as medians and interquartile range (25‒75).

BMI, Body Mass Index; AC, Abdominal Circumference; CC, Cervical Circumference; SBP, Systolic Blood Pressure; DBP, Diastolic Blood Pressure.

**Table 4:** Comparison of groups with and without apnea regarding polysomnographic variables.

Variable	AHI < 5 (*n* = 11)	AHI ≥ 5 (*n* = 19)	p
TTS (Min)	361.08 ± 62.55	313.02 ± 70.19	0.070
Eficiency ( %)	78.24 ± 14.30	69.87 ± 14.31	0.133
% TTS REM	12.01 ± 5.42	9.2 ± 6.91	0.256
Respiratory events and sleep stages (Index/hour)			
AHI REM	2.3 (0.5; 15.45)	50.7 (30.2; 65.40)	**<0.001**
AHI NREM	1.3 (0.8; 1.6)	17.9 (7.75; 39.45)	**<0.001**
Hipopneas REM	2.1 (0; 13.85)	17.8 (4.7; 23.65)	**0.042**
Hipopneas NREM	1.2 (0.5; 1.6)	9.9 (6.75; 19.35)	**<0.001**
Obstructive apneas REM	0 (0; 1.6)	21.1 (5.2; 48.5)	**0.003**
Obstructive apneas NREM	0 (0; 0.2)	3.5 (1.3; 13.4)	**<0.001**
AHI supine position	2.2 (0.3; 4)	26 (9.95; 45.95)	**<0.001**
Time in supine ( %)	60 (10.5; 89.6)	85 (60.05; 100)	0.169
AHI non supine position	1.1 (0.9; 1.8)	6.1 (1.5; 13.3)	0.082
Time in non-supine ( %)	40 (10.35; 89.5)	15 (0; 39.95)	0.127

Parametric variables expressed as means ± standard deviation. Non-parameric variables expressed as medians and interquartile range (25‒75).

TTS, Total Sleep Time; AHI, Apnea Hipopnea Index; REM, Rapid Eye Moviment.

Concerning endocrine-metabolic variables, there was an association between the presence of obstructive sleep apnea and diagnoses of glycemic alterations and low HDL cholesterol. Furthermore, the population with OSA also presented higher mean values of systolic blood pressure when compared to subjects without OSA, also with a large effect size (g Hedge = 0.85). Although not statistically significant, subjects with OSA had higher frequencies of daytime naps and a higher average index on the Epworth Daytime Sleepiness Scale when using an AHI cutoff point ≥ 5. From a functional point of view, there was no difference in performance indices for basic and instrumental activities of daily living when comparing these groups.

In a comparison between groups with and without OSA, no differences were observed in polysomnographic variables, including total sleep time, sleep efficiency, percentage of time spent in REM sleep, and percentage of recording time in supine and non-supine positions. Subjects with OSA presented apneas associated with the supine position (Table 4).

The group with AOS showed worse performance in tests evaluating episodic and semantic verbal memory (*p* = 0.022) with a large effect size (g Hedge = 1.219) and in the recognition subitem (*p* = 0.038) (Table 5).

**Table 5:** OSA × non-OSA: Cognitive assessment.

	AHI < 5 (*n* = 6)	AHI ≥ 5 (*n* = 14)	p
Age	21.5 (21; 22.75)	24 (21.5; 27.75)	0.41
Literacy ( %)	66.7	57.1	>0.999
Barthel index	100 (100; 100)	97.5 (90; 100)	0.122
Lawton	15.66 ± 2.87	15.64 ± 4.27	0.990
QIT	48.16 ± 4.58	45.21 ± 3.33	0.121
QIV	51.16 ± 5.31	49 ± 3.44	0.287
QIE	54.66 ± 4.84	52.21 ± 5.59	0.363
Code	3 (3; 3.75)	3 (3; 4.5)	0.467
Digit	4 (2; 4)	3.5 (2; 4)	0.883
Windows	1 (1; 1)	1 (1;1)	0.894
Simbols	5 ± 1.41	4 ± 1.57	0.196
Memory	−3.33 ± 2.61	−3.45 ± 1.33	0.915
Work memory	−2.56 ± 1.92	−2.24 ± 1.50	0.689
A) Digit spam	−4.47 (−4.49; −3.51)	−2.57 (−4.60; −1.90)	0.706
B) Word spam	−2.74 (−2.98; −0.52)	−1.80 (−2.73; −0.7)	0.772
V. E. S. memory	−1.57 ± 1.69	−3.13 ± 1.08	**0.022**
A) Immediate recall	−1.40 ± 1.56	−2.40 ± 0.75	0.184
B) Delayed recall	−0.93 (−1.41; 0.63)	−1.30 (−1.63; −1.27)	0.319
C) Recognition	−1.045 (−2.55; −0.46)	−5.58 (−5.78; −2.8)	**0.038**
Attention	−4.93 ± 1.31	−3.64 ± 1.77	0.128
Counting backwads (time)	−4.92 ± 1.64	−3.66 ± 2.19	0.225
Reverse digit spam	−1.705 (−1.71; −1.70)	−1.700 (−1.74; −1.7)	0.544

Parametric variables expressed as means ± standard deviation. Non-parameric variables expressed as medians and interquartile range (25‒75).

QIT, Total Intelligence Coefficient; QIE, Execution Intelligence Coefficient; QIV, Verbal Intelligence Coefficient; V. E. S., Episodic and Semantic Verbal Memory.

A comparison was made between the subgroup that underwent cognitive performance analysis (*n* = 20) and the subgroup that was excluded (*n* = 10) due to clinical reasons, according to the loss flowchart. Age was the only variable with a statistically significant difference (Cognitive group: mean age 24.65-years vs. 30.2, *p* = 0.031). There were no significant differences between the groups with respect to schooling (9 vs. 9.5 years, *p* = 0.941), literacy (*p* = 0.442), the presence of sleep apnea (*p* = 0.283), Barthel Index (*p* = 0.479) and Lawton Scale (*p* = 0.259).

**Fig. 2:**
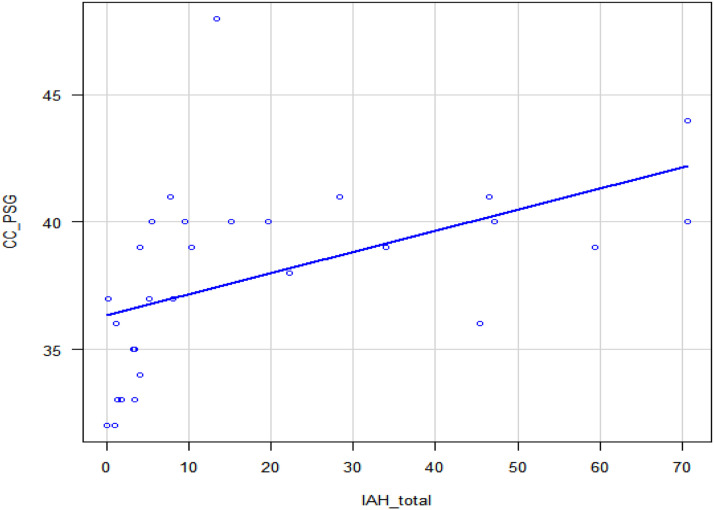
AHI and CC.

**Fig. 3:**
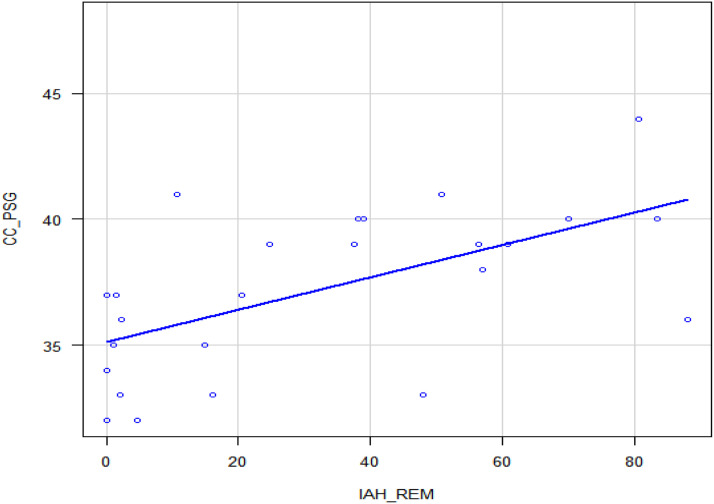
AHI-REM and CC.

There was a strong correlation (*r* = 0.731) between total AHI and cervical circumference, *p* < 0.001 (Table 6, Fig. 2) and also in REM sleep (Table 6, Fig. 3). No statistically significant correlations were found between the AHI in REM sleep and the other body composition variables used in this study. This analysis included all individuals, with and without a diagnosis of Obstructive Sleep Apnea (OSA), without initial stratification by sex.

**Table 6:** AHI and body composition correlation.

Variável	AHI total		AHI REM sleep	
	Coeficiente	p	Coeficiente	p
BMI	0.199	0.291	0.006	0.975
CC	0.731	**<0.001**	0.58	**0.002**
AC	0.339	0.071	0.253	0.222
AC/height	0.147	0.446	0.030	0.884

BMI, Body Mass Index; AC, Abdominal Circumference; CC, Cervical Circunference.

In a post hoc gender stratified analysis, among women, CC maintained a significant positive correlation with AHI (*r* = 0.737; *p* = 0.001), while among men, it was not significant (*r* = 0.089; *p* = 0.761). Multiple linear regression analysis, including the interaction between CC and sex, confirmed that CC was a significant predictor of AHI in women (β = 4.01; *p* = 0.023), but not in men, and the CC × sex interaction was not significant (*p* = 0.137).

## Discussion

This study demonstrated the high frequency of several clinical comorbidities in adults with DS, similar to international studies, in an outpatient clinic with longitudinal follow-up and with a comprehensive look at their needs.^4^, ^5^, ^6^, ^7^ Performance in carrying out basic activities of daily living was very low in dependency in the studied population. However, they showed moderate dependence in carrying out instrumental activities of daily living, measured by the Lawton scale. The prevalence of obstructive sleep apnea was high, with 63 % frequency. The group with AHI ≥ 5 events/hour, when compared to the group without OSA, was associated with male sex, greater cervical circumference, greater deposition of abdominal fat, and worse performance in verbal episodic and semantic memory, in addition to higher mean systolic blood pressure, higher frequency of diagnoses of glycemic changes and lower HDL cholesterol. Cervical circumference correlated strongly and statistically significantly with total AHI.

People with DS are at greater risk of upper airway collapse due to craniofacial anatomical changes including maxillary and midface hypoplasia, relative macroglossia due to mandibular hypoplasia, in addition to its hypotonia.^14^ Besides these anatomical changes, there is the high prevalence of obesity and hypothyroidism in this population, which are risk factors for OSA described in the general population. The present study found an association between OSA and the male sex, extending what has been demonstrated in prevalence studies in the general population.^22^ The studied population with OSA also had greater cervical and abdominal circumference when compared to subjects without OSA (40 vs. 34 cm; 92.6 vs. 82.5 cm, respectively). The greater volume of the tongue and lateral walls of the pharynx were predictors of a greater risk of OSA in a case-control study using magnetic resonance imaging to evaluate soft tissue structures of the upper airways.^23^ Furthermore, greater tongue volume was associated with variables related to obesity (BMI, abdominal and neck circumferences) in a cross-sectional study with 34 adults using computed tomography.^24^ The authors found a strong and significant positive correlation between AHI and cervical circumference (*r* = 0.731). The centripetal deposition of fat, measured by abdominal circumference, implies greater deposition of fat in the cervical region, possibly contributing to the collapsibility of the airways. Among the OSA patients in this analysis, 73 % were male and their BMI was lower than that of females. This difference may explain the lack of association, in this study, between BMI and AHI.

OSA is associated with metabolic and cardiovascular comorbidities, including systemic arterial hypertension and metabolic syndrome.^25^^,^^26^ In the present study, the group with AHI ≥5 events/hour, when compared to the group without OSA, presented higher mean levels of systolic blood pressure, higher frequency of diagnoses of glycemic changes and lower HDL cholesterol, throughout the five-year follow-up. Episodes of apnea and hypopnea contribute, through hypercapnia and hypoxemia, to the activation of the sympathetic system and elevation of catecholamine levels, with consequent elevation of blood pressure, in addition to other pathophysiological phenomena associated with hypoxia, including oxidative stress, endothelial dysfunction, as well as the activation of the renin-angiotensin-aldosterone system, which together contribute to the cardiovascular consequences of OSA.^25^

Neurocognitive impairments are well described in systematic reviews and meta-analyses in the general population with OSA, including deficits in executive functions, attention and memory.^27^, ^28^, ^29^ Recent studies demonstrate that OSA may be a modifiable risk factor for cognitive decline.^30^^,^^31^ In the present study, the group with AHI ≥5 events/hour, when compared to the group without OSA, showed worse performance in episodic verbal and semantic memory, measured by the Brazilian Brief Assessment Instrument NEUPSILIN. To the best of our knowledge, there are few studies on cognitive outcomes in people with DS and OSA, especially in the adult population. The effect size observed for memory deficits was large (*g* = 1.219), suggesting a practically relevant difference. However, given the small sample size, these findings should be interpreted with caution and considered as preliminary, reinforcing the need for replication with greater statistical power.

Among the general adult population, subjects with OSA show worse performance in episodic memory tests in the verbal domains of immediate, delayed recall, and recognition, compared to healthy controls.^29^ The present study extended to adults with DS and untreated OSA, the worst performance in verbal episodic memory found in studies carried out in adults without neurodevelopmental alterations, and in its subdomain of recognition.^29^^,^^32^ The analysis of cognitive performance showed that the groups did not differ in terms of age and education, nor in terms of literacy percentage, factors that can alter performance in the NEUPSILIN neuropsychological assessment.^33^

No differences were found in attention and executive functions between subjects with and without OSA in this study, unlike several studies on people without DS. People with DS have deficits in episodic memory, long-term explicit memory, executive functions, including: working memory, inhibitory control, set shifting, planning and resolution of problems, sustained attention. The type of cognitive deficit and its severity vary between individuals.^34^^,^^35^ Furthermore, the great variation between the cognitive tests used and their “floor effects” stands out in studies carried out with this population.^36^ According to Spaniol et al., studies are heterogeneous as regards the definition of cognitive abilities included under the name of executive functions, and there is great variability of the tests used to assess them.^37^ Also, impairments in alertness, vigilance, and attention may affect performance on other cognitive tests, including executive functions, as demonstrated by Verstraeten et al.^38^

There are few studies on cognitive outcomes in people with DS and OSA, especially in the DS adult population, as evidenced in the review by Gandy et al. In the present study, no significant differences were found in the tests that assessed IQ, executive functions, working memory and attention, possibly due to floor effects in their performance and small sample size. Besides, the level of attention may have contributed to the lack of difference in executive functions since they were strongly correlated (Correlation between digit span and attention: *r* = 0.69; *p* = 0.0007).

The present study has limitations that include its exploratory nature, small sample size, and selection bias related to convenience sampling. The small sample size limits statistical power, especially for subgroup analyses, and the convenience sample (from a tertiary center that excluded subjects with decompensated comorbidities) limits external generalization and may underestimate the severity of OSA. The study population was young, and half of them were literate, which may compromise generalization to older populations or those with less education. However, few studies evaluated the prevalence of OSA in adults with DS, most of them with a small number of participants or using a questionnaire applied to family members or main caregivers as a method of assessing the presence of OSA. Besides, this is a poorly studied population with highly variable clinical characteristics, which makes it difficult to conduct studies with large numbers of participants and samples that are homogeneous in terms of their clinical characteristics.

As regards methodological issues, the cross-sectional design makes it difficult to draw cause and effect of the results. Another important limitation of the study concerns the cognitive assessments. Only 20 participants were included in the analysis of cognitive variables. This subgroup was compared to the subgroup excluded due to clinical variables. Although these groups presented similar demographic and functional characteristics, with the exception of age, it is possible that there is attrition bias due to the presence of variables not included in the model. Also, NEUPSILIN’s normative data may not fully account for DS-specific cognitive profiles.

This pilot exploratory study was performed in search of possible clinical associations, to generate insights for future studies. The authors used an objective test to diagnose OSA, a complete polysomnography. Also, over a 5-year follow-up, the authors carried out periodic health assessments that allowed us to clinically characterize, in detail, the study population. Another strength herein, is that the possible health consequences of OSA, common among the general population, including metabolic and cognitive variables, which are little explored in the literature in adults with DS, were researched with objective tests, including cognitive assessment, functional performance with scales validated in Brazil, including the Barthel and Lawton scales.

In conclusion, the present study, which included a five-year follow-up of young adults with Down syndrome, showed a high prevalence of multimorbidity, including specific ophthalmological and otorhinolaryngological pathologies, such as keratoconus and hearing loss, lipid changes, overweight, obesity, hypothyroidism, celiac disease, gastroesophageal reflux disease, renal lithiasis, cervical instability and obstructive sleep apnea. These results are preliminary and reinforce the need for studies with larger samples to provide clinical recommendations for this population. The authors also identified a potential association between OSA and both metabolic profile and memory performance. Considering that individuals with Down syndrome present a high risk of early-onset dementia, the coexistence of OSA and its possible link to poorer metabolic and cognitive outcomes may hold considerable clinical relevance. These findings underscore the need for further research in this area, particularly studies exploring therapeutic interventions targeting OSA and obesity.

## Ethics approval statement

The study was approved by the Research Ethics Committee of the Hospital das Clínicas of the University of São Paulo (CAAE: 23,969,319.8.0000.0068 number 3.686.083).

## Authors’ contributions

LFI, MAM, PZT: Conceptualization; Data curation; Methodology; Analysis; Writing and review.

PPBCC: Writing; Review and editing.

CCAR: Conceptualization and analysis of cognitive assessments; Review and editing.

RH: Conceptualization; Analysis of polysomnography’s; Review and editing

GLF, OVF: Conceptualization; Review and editing.

## Funding

This study has received nonfinancial support.

## Data availability

The datasets generated and/or analyzed during the current study are available from the corresponding author upon reasonable request.

## Acknowledgments

This report is part of a the first author's doctoral thesis developed by the first author, under the guidance of the last author, at the University of São Paulo. The authors would like to thank the individuals with Down syndrome and their families for agreeing to participate in the study. The authors also would like to acknowledge: Gisele Correa de Moraes, Priscila Ribeiro, Natalia Jansen, Beatriz Cristina Filliettaz, who did the cognitive evaluation of the study population.

## Declaration of competing interest

The authors declare no conflicts of interest.

## Appendix Supplementary materials

Image, application 1
