# Supplementary material for: Obstructive sleep apnea in adults with Down syndrome: body composition, metabolic profile and cognitive status
Source: Clinics (Sao Paulo). 2026 Feb 13;81:100867. doi: 10.1016/j.clinsp.2026.100867 (PMC12924187; doi:10.1016/j.clinsp.2026.100867)
Supplement: Supplementary file 1 [file mmc1.docx]

**Supplementary Material**

Regarding the presence of apnea and cognitive performance, there was a weak to moderate negative correlation between episodic verbal memory performance and total AHI, as well as its sub-items immediate recall and recognition memory, with marginal results in the two-tailed analysis.

Correlation AHI and cognitive performance (n = 20).

| **Variable** | **Total AIH** | | **REM sleep AHI** | |
| --- | --- | --- | --- | --- |
|  | **Coeficient** | **p** | **Coeficient** | **p** |
| QIT | -0.177 | 0.456 | -0.249 | 0.335 |
| QIV | -0.082 | 0.731 | -0.095 | 0.715 |
| QIE | -0.131 | 0.583 | -0.199 | 0.444 |
| Memory | -0.114 | 0.633 | -0.185 | 0.477 |
| Work memory | -0.095 | 0.688 | -0.168 | 0.519 |
| A) Digit spam | -0.12 | 0.615 | -0.106 | 0.685 |
| B) Word spam | -0.066 | 0.781 | -0.058 | 0.824 |
| V. E. S. memory | -0.367 | **0.112** | -0.263 | 0.307 |
| A) Immediate recall | -0.389 | **0.089** | -0.236 | 0.361 |
| B) Delayed recall | -0.332 | **0.152** | -0.327 | 0.2 |
| C) Recognition | -0.429 | **0.0589** | -0.203 | 0.434 |
| Attention | 0.148 | 0.535 | 0.278 | 0.28 |

QIT, Total Intelligence Coefficient; QIE, Execution Intelligence Coefficient; QIV, Verbal Intelligence Coefficient; V. E. S., Episodic and Semantic Verbal memory.
